# Human Chorionic Gonadotropin and Breast Cancer

**DOI:** 10.3390/ijms18071587

**Published:** 2017-07-21

**Authors:** Susanne Schüler-Toprak, Oliver Treeck, Olaf Ortmann

**Affiliations:** Department of Obstetrics and Gynecology, University Medical Center Regensburg, Caritas-Hospital St. Josef, 93053 Regensburg, Germany; sschueler@caritasstjosef.de (S.S.-T.); oortmann@caritasstjosef.de (O.O.)

**Keywords:** human chorionic gonadotropin, pregnancy, breast cancer

## Abstract

Breast cancer is well known as a malignancy being strongly influenced by female steroids. Pregnancy is a protective factor against breast cancer. Human chorionic gonadotropin (HCG) is a candidate hormone which could mediate this antitumoral effect of pregnancy. For this review article, all original research articles on the role of HCG in breast cancer were considered, which are listed in PubMed database and were written in English. The role of HCG in breast cancer seems to be a paradox. Placental heterodimeric HCG acts as a protective agent by imprinting a permanent genomic signature of the mammary gland determining a refractory condition to malignant transformation which is characterized by cellular differentiation, apoptosis and growth inhibition. On the other hand, ectopic expression of β-HCG in various cancer entities is associated with poor prognosis due to its tumor-promoting function. Placental HCG and ectopically expressed β-HCG exert opposite effects on breast tumorigenesis. Therefore, mimicking pregnancy by treatment with HCG is suggested as a strategy for breast cancer prevention, whereas targeting β-HCG expressing tumor cells seems to be an option for breast cancer therapy.

## 1. Introduction

Breast cancer is the most common malignancy in women worldwide, with more than 1.5 million new cases diagnosed every year; it is the second most common cancer overall. Every year, about half a million women die of this disease [1]. For over a hundred years, since the regression of metastatic breast cancer after an oophorectomy was reported, it has been recognized that this tumor entity is strongly influenced by hormonal factors. Estrogens were the first sex hormones identified to promote breast cancer growth via binding to the nuclear estrogen receptor α [2]. Further reports indicated that levels of most sex steroid hormones are strongly associated with breast cancer risk [3]. With regard to reproductive peptide sex hormones, prolactin plasma levels have previously been reported to affect the risk of breast cancer development in postmenopausal women [4].

## 2. Results

### 2.1. Pregnancy and Breast Cancer

Beside other risk factors of breast cancer, like patient’s age, family history of breast cancer, early menarche, late menopause, high body mass index, socioeconomic status, ethnicity and a genetic predisposition for breast cancer, the reproductive history of women was shown to affect breast cancer risk [5,6]. A pregnancy, particularly under the age of twenty, exerts a strong protective effect against this disease [7]. When assessing the effect of parity on prognosis of breast cancer patients, not only the parous status but also the number of childbirths and the age at first delivery have to be taken into account [8]. However, many reports on these topics are highly controversial. Concerning the role of parous status on breast cancer prognosis, some studies found the best prognosis to be associated with nulliparity [9,10,11]. In contrast to these observations, two studies reported a better breast cancer prognosis of parous women [12,13]. However, other studies, including the largest on the subject by Kroman et al., did not find a prognostic importance of the status as nulliparous versus parous [14,15,16,17,18]. The number of deliveries was positively correlated with poor prognosis in some studies [9,10,11,14,19,20,21]. A recent large study including 1140 invasive breast cancer patients, reported that high parity (3+ births) was associated with breast cancer-specific mortality, independent of age, race, and selected socioeconomic factors (adjusted hazard ratio (HR) = 1.76; 95% confidence interval (CI) = 1.13–2.73). The association was stronger among patients with luminal tumors and those surviving longer than 5 years [21]. Though other studies did not find a correlation, and no study reported the opposite correlation, the adverse effect of high parity on breast cancer prognosis is better documented than the effect of the parous state alone [12,13,16,17]. It is much less controversial that younger women have a worse prognosis compared to women who postpone childbearing to a later age, although the risk of younger women getting breast cancer is reduced [15,17,18,20,22]. Pregnancy-associated breast cancers (PABC), those diagnosed during pregnancy, lactation, or in the first postpartum year, are typically found at an advanced stage, have a higher incidence of lymph node metastases and are poorly differentiated [22,23,24,25,26,27,28,29,30]. As a consequence, diagnosis of breast cancer shortly after pregnancy was associated with reduced survival in several studies [8,10,20,21,22,31,32,33,34,35]. In contrast, a large retrospective analysis showed that prognosis of young patients with PABC is not statistically significantly different compared with those with non-PABC [30]. 

However, the influence of breastfeeding also has to be assessed when evaluating the effect of pregnancy on breast cancer risk. Several studies showed an inverse correlation of breast feeding with breast cancer risk [36]. In the “Cancer and Steroid Hormone Study” which included 4599 women between 22 and 50 with a diagnosis of breast cancer and 4536 controls, and women who had breastfed for 25 months or more had an adjusted relative risk (RR) of 0.67 (95% CI 0.52–0.85) compared to parous women who never breastfed [37]. The “Collaborative Group on Hormonal Factors in Breast Cancer” evaluated individual data from 47 epidemiological studies in 30 countries that included information on breastfeeding patterns and other aspects of childbearing for 50,302 women with invasive breast cancer and 96,973 controls. Women with breast cancer had, on average, fewer births than did controls (2.2 vs. 2.6). Furthermore, fewer parous women with cancer than parous controls had ever breastfed (71% vs. 79%), and their average lifetime duration of breastfeeding was shorter (9.8 vs. 15.6 months). Also, the duration of lactation seems to influence breast cancer risk. The RR of breast cancer decreased by 4.3% (95% CI 2.9–5.8; *p* < 0.0001) for every 12 months of breastfeeding in addition to a decrease of 7.0% (5.0–9.0; *p* < 0.0001) for each birth [38]. Zhou et al. performed a meta-analysis including 27 studies involving 13907 breast cancer cases and found a RR of breast cancer for the ever compared with never categories of breastfeeding was 0.613 (95% CI 0.442–0.850). An inverse association was also found for the longest compared with the shortest categories of breastfeeding with the risk of breast cancer (RR 0.471; 95% CI 0.368–0.602) [39]. However, the risk reduction by lactation differs between the different breast cancer subtypes. Lambertini et al. performed a systematic review and meta-analysis and found that having ever-breastfed was associated with a reduced risk of developing both luminal (pooled odds ratio (pOR) 0.77; 95% CI, 0.66–0.88; *p* = 0.003) and triple-negative (pOR 0.79, 95% CI, 0.66–0.94; *p* = 0.01) subtypes [40]. This is in line with the data published by Islami et al. showing that in a subset of three studies that adjusted for estrogen receptor (ER), progesterone receptor (PR), and human epidermal growth factor receptor 2 (HER-2/neu) status, having ever breastfed showed a stronger inverse association with triple-negative breast cancer (odds ratio (OR) 0.78; 95% CI 0.66–0.91) among parous women [41]. However, these findings are still discussed controversially.

### 2.2. CGB Genes, Heterodimeric Human Chorionic Gonadotropin and Its Receptor in Breast Cancer

The pregnancy hormone human chorionic gonadotropin (HCG) is a complex glycoprotein with a molecular mass of about 37 kDa. The heterodimer consists of two different glycosylated subunits, the α-subunit being typical for the glycoprotein protein family, and a receptor-specific β-subunit [42]. 

The heterodimeric form of HCG is primarily produced in placental syncytiotrophoblasts and trophoblastic tumors. Beside the main sites of expression, the placenta, *β-HCG* genes are also expressed in normal non-trophoblastic tissues, mostly in testis, prostates, thymus, skeletal muscles and pituitary glands [43,44,45]. Its role in normal tissue seems to be mainly autoregulatory exerting growth-promoting activity [46]. However, the β-subunit of HCG, and to a lesser extent heterodimeric HCG, are also produced by several non-trophoblastic tumors like breast cancer, cervical carcinoma, vaginal cancer, bladder cancer, lung cancer, colorectal carcinoma, prostate cancer and gastric cancer [44,47].

The HCG β-subunit is encoded by a cluster of genes (chorionic gonadotropin beta *(CGB)*) and contains several glycosylation sites. The location of the luteinizing hormone beta polypeptide *(LHB)/CGB* gene cluster is a dynamic gene-rich region. The structure and sequence content of the human *LHB/CGB* gene cluster represents a typical young genomic region evolved by duplication events. It consists of one *LHB* gene, four β-HCG coding genes *(CGB, CGB5, CGB8* and *CGB7)* and two gene copies with unknown function (*CGB1* and *CGB2*) [44,48,49,50]. *LHB* genes situated in the pituitary gland are limited in their rate of hormone production by transcription and are kept in a narrow frame. Contrarily, placental β-HCG mRNA is expressed by four duplicate genes exhibiting high genetic variation [44]. Larger inter-individual differences in the total amount of produced mRNA and in hormone levels are tolerated than in the case of pituitary gonadotropins [51,52]. The coaction between gene conversion and selective forces drives its evolution and it is one of the most polymorphic genes in the human genome. 

The prerequisite for action of placental HCG on mammary tissue is the presence of its specific luteinizing hormone (LH)/HCG receptor. This receptor, which also binds LH, is primarily located in the ovarian follicle, on ovarian corpus luteum cells for promotion of progesterone production and on trophoblast, cytotrophoblast and syncytiotrophoblast cells for enhancement of trophoblast cell differentiation and for immunosuppression and macrophage suppression for foreign invading cells. Both in the mammary epithelium and in many breast cancer cases, LH/HCG receptors are also expressed. In normal mammary tissue, their expression was found to increase in the luteal phase of the menstrual cycle. LH/HCG receptor expression was reported to be higher in normal breast tissue than in breast cancer [53,54], suggesting that the effect of HCG on normal breast tissue might be more pronounced than its effect on breast cancer tissue. In breast cancer tissue, the correlation of the LH/HCG receptor expression with tumor characteristics remains unclear. Whereas in one study, expression of this receptor was reported to be associated with favorable tumor characteristics, in a conflicting study it has been reported to be increased in invasive breast cancer cases when compared to preinvasive specimen [53,55]. 

It is quite interesting that several single-nucleotide polymorphisms (SNPs) have been found in gonadotrophins and their receptor genes, resulting in several genotypes that are differently distributed among human populations. They modulate signal transduction and affect sex-related reproductive features [56,57]. The *LH/HCG receptor* gene (*LHCGR)* carries at least 300 known polymorphisms, however, only a few of them have relevant effects [56]. One of them, the *LHCGR variant 18insLQ,* is associated with the early onset of breast cancer and short disease-free survival. This is consistent with increased sensitivity and plasma membrane expression of this receptor variant (1.9-fold lower HCG half-effective concentration and 1.4-fold higher expression levels than wild type (*WT) LHCGR*, respectively; [58]). This results in a higher LH/HCG receptor activity. Probably by increasing estrogen exposure, breast cancer disease-free survival is thereby shortened [58]. Interestingly, *LHCGR 18insLQ* has a high frequency among Northern-European Caucasians that are characterized by a higher prevalence of breast cancer compared to other ethnic groups, leading to the speculation that the *LHCGR* genotype may be linked to disease risk [56,57,59].

There are several theories about the role of HCG in carcinogenesis. Trophoblasts use the biological means of HCG for successful implantation and placentation. Moreover, activation of transcription of *CGB*, *CGB5* and *CGB8* genes has been associated with malignant transformation of non-trophoblastic cells [43,44,60,61]. 

The HCG β-subunit glycosylation state varies with the stage of pregnancy, its source of production and the pathology. For years, the main role of HCG was seen in its luteotrophic effect on the maternal corpus luteum. However, it is now understood that HCG is not a single entity. There are five independent variants including the “classic” HCG, acting as a hormone, the sulphated HCG, exerting hormone-like effects, and hyperglycosylated HCG (HCG-H), acting as an autocrine signaling factor [62]. HCG-H has double-sized O-linked oligosaccharides and extra-large N-linked oligosaccharides, which makes the molecule larger and more acidic [62,63]. It has been determined that HCG-H is predominant during the implantation stages of pregnancies as well as in gestational choriocarcinoma and testicular cancer malignancy [64,65,66,67]. Β-HCG-large has recently been demonstrated to be a hyperglycosylated β-subunit (β-HCG-H), with a similar larger sugar structure to that of HCG-H [68]. Research shows that β-HCG and β-HCG-H, as produced by cancer cells, are also autocrine growth factors, directly promoting cancer cell invasion, cancer cell growth and metastases [43,62,69,70,71].

The majority of previous studies clearly suggests a protective or inhibitory action of heterodimeric placental HCG on breast cancer development, though other studies could not find such an effect. In animal models, administration of HCG reduced risk of carcinogen-induced breast cancer, an effect which was mediated by induction of apoptosis and elevated inhibin expression [72,73,74]. Various studies reported a negative correlation between serum HCG levels and breast cancer risk. Russo et al. found that high levels of HCG during the first weeks of pregnancy reduced the incidence of maternal breast cancer rate after the age of 50 [75]. Toniolo et al. reported a risk reduction of about 30% of getting breast cancer when women had higher HCG levels in the first three months of pregnancy [76]. Lukanova et al. found that women with HCG levels in the top tertile tended to be at lower risk of breast cancer than women with HCG levels in the lowest tertile [77]. No such inverse relationship between early pregnancy serum HCG concentrations and breast cancer risk was found by other studies including in a recent large prospective study from Finland including 1191 women with invasive breast cancer and 2257 controls [78]. The beneficial effect of HCG was supported by the results of two studies examining the effect of treatment with recombinant HCG. In a placebo-controlled study, post-menopausal women with primary breast cancer diagnosed by core biopsy were treated with HCG or a placebo for two weeks. After this period, therapeutic mastectomies or lumpectomies were performed. Tissue examinations demonstrated that the proliferative cell index was decreased from 18 to 4% in the HCG-treated group, suggesting that this hormone is able to inhibit breast cancer cell proliferation [79]. In another study, young nulliparous women receiving injections of HCG for weight loss or infertility treatment were reported to have a slightly decreased risk for developing breast cancer [80]. 

### 2.3. Heterodimeric HCG Induces Differentiation and Apoptosis 

At the cellular and molecular level, placental HCG was reported to exert a differentiating, pro-apoptotic and growth-inhibitory effect on breast epithelial and breast cancer cells. In a rat model, HCG prevented the development of chemically-induced tumors by inducing differentiation of the mammary gland, an effect accompanied by increased expression of tumor suppressor inhibin, downregulation of estrogen receptor α (ER α) expression by methylation of 5′-C-phosphate-G-3′ (CpG) islands, imprinting a permanent genomic signature characterizing the refractory condition of the mammary gland to undergo malignant transformation. This genomic signature induced by HCG was identical to that induced by pregnancy and is specific for this hormone [81]. In MCF-7 breast cancer cells, HCG induced expression of differentiation markers β-casein, cytokeratin-18 and E-cadherin [82], also supporting an antitumoral role of this hormone. The in vitro study performed by Rao et al. demonstrates that the antiproliferative effects of HCG in MCF-7 cells could be induced by downregulation of nuclear factor kappa B (NF-κB) and AP-1 [83]. A pro-apoptotic function of this hormone has also been reported in a convincing study where breast cancer cell-transplanted athymic mice where treated with HCG or saline for 11 weeks and resulted in a extreme reduction of tumor growth triggered by the hormone. The most pronounced effect on growth reduction was achieved with KPL-1 cells. Immunoblotting analysis revealed a peak of tumor suppressor protein p53 12 h after treatment, followed by a cleavage of caspase-9 and -3. In vivo, an elevated estrogen levels were observed after five weeks [84]. This observation is interesting, as these high estrogen levels could oppose the antiapoptotic effect in other models.

The differentiating effects of heterodimeric HCG are accompanied by growth-inhibitory and pro-apoptotic actions on breast epithelial and breast cancer cells. In a recent study, HCG was reported to reduce the proliferation of MCF-7 breast cancer cells by decreasing the expression of proliferating cell nuclear antigen (PCNA) and proliferation-related Ki-67 antigen (Ki-67) [82]. In the same cell line, HCG treatment led to increased expression of genes involved in apoptosis and DNA repair, but to a decrease of proliferation gene expression [85]. In normal breast epithelial cells, HCG was reported to activate expression of the apoptotic and growth inhibitory genes transient receptor potential cation channel subfamily M member 2 *(TRPM2)*, *ICE*, transforming growth factor β *(TGF-β)*, *p53*, BCL2 associated X, apoptosis regulator *(BAX)*, and cyclin-dependent kinase inhibitor p21 *(p21WAF1)* [86]. Injection of HCG into breast cancer xenografts grown in nude mice was reported to increase the apoptotic index of the cells [87]. The apoptotic effect of HCG on breast cancer cells has been shown to be mediated by activation of the p53 mitochondrial apoptotic pathway leading to cleavage of caspases 3 and 9 [84] (Table 1).

### 2.4. Ectopically Expressed β-HCG in Breast Cancer

HCG and particularly its subunit β-HCG are known to be ectopically produced by several non- trophoblastic carcinomas. Ectopic expression of HCG is seen in poorly differentiated and high grade tumors and particularly tissue and serum levels of ectopic β-HCG are associated with poor prognosis [88,89,90,91,92,93,94]. Early reports indicated that malignant transformation of various tissues including the breast is associated with acquired expression of genes coding for the β-subunit of HCG, which are normally expressed in trophoblastic cells only. The *β-HCG* genes *CGB3*, *CGB5*, *CGB8* and *CGB9*, called type II β-HCG genes, were found to be expressed in 46% of breast cancer cases, but not in normal mammary epithelium [43]. The β-HCG subunit, which can be present in cancer cells as free β-HCG or as β-HCG homodimers, has been shown to exert effects on tumorigenesis totally different from heterodimeric placental HCG. β-HCG has been suggested to be part of the TGFβ oncoprotein family of molecules [95,96]. Free β-HCG and β-HCG homodimers have been reported to act in autocrine manner and bind to TGFβ receptors on the producing cell, thereby antagonizing the antitumoral action of these receptors. Thus, β-HCG has been suggested to exert tumorigenic actions by blocking the apoptotic effect of TGFβ1 in cancer cells of different origin by TGFβ receptor binding [46,62,70]. However, activation of TGFβ receptors by this hormone remains controversial, because it might result from contamination of HCG preparations with epidermal growth factor (EGF) or TGFβ1 [97]. Confirming the anti-apoptotic effect of β-HCG, RNA interference (RNAi)-mediated knockdown of this subunit in cervical cancer cells led to induction of apoptosis [98]. Hyperglycosylated HCG and free β-HCG were suggested to be interchangeable cancer promoters functioning by antagonizing TGFβ receptor. It is thought that hyperglycosylated HCG produced in early pregnancy promotes implantation by the same mechanism of tumorigenesis, involving blockage of apoptosis and promotion of metalloproteinase and collagenase production [99,100].

Besides its anti-apoptotic action, ectopically expressed β-HCG has been shown to promote invasion by down-regulation of E-cadherin, which plays a crucial role in the epithelial cell to cell adhesion and is an invasion suppressor [101]. β-HCG was also reported to increase invasiveness and motility of cancer cells of different origin by activation of extracellular signal–regulated kinase (ERK)1/2 and matrix metalloprotease (MMP)-2 [102,103,104].

Thus, autocrine inhibition of apoptosis and activation of invasion triggered by β-HCG might be the main mechanisms underlying the adverse effect of *β-HCG* gene expression in breast cancer cells, which has been reported by several studies. Expression of β-HCG mRNA was reported as a useful marker for detection of metastatic breast cancer cells in whole blood and in lymph nodes, being positive in 80% of blood samples from breast cancer patients and in 61% of tumor-draining axillary nodes [105]. The combination of β-HCG and tumor antigen MAGE family member A3 (MAGE-A3) expression in circulating breast cancer cells positively correlated with tumor size [106]. High expression of *CBG 3*, *5* and *8* in breast cancer has been reported to have an independent prognostic value for short relapse-free survival (RFS) [107]. Finally, transgenic mice overexpressing β-HCG developed multiple neoplasms including breast cancer which was accompanied by dysregulation of wnt signaling in the mammary gland [108]. Thus, expression of β-HCG can not only be used for detection of breast cancer cells, but also seems to correlate with adverse parameters (Table 1).

### 2.5. Immunological Approaches Directed to β-HCG

The cancer promoting function of ectopically expressed β-HCG in tumor cells was the basis for development of immunological approaches against this subunit as a possible therapeutic strategy for treatment of β-HCG expressing cancer like breast cancer. One of these approaches is vaccination against β-HCG. In previous studies, the effect of different β-HCG vaccines on tumor growth has been tested. With regard to breast cancer, a combined vaccination with β-HCG bound to a heat shock protein and a β-HCG DNA vaccine was reported to effectively inhibit the growth of breast cancer cells in a mouse model [109]. A synthetic vaccine targeting β-HCG composed of the terminal peptide of β-HCG conjugated to diphtheria toxoid (DT) induced anti β-HCG antibodies in patients with advanced colorectal cancer, which was associated with longer overall survival. Patients with higher levels of anti β-HCG antibodies exhibited a median survival of 45 weeks compared with 24 weeks for patients who developed lower levels of this antibody (*p* = 0.0002) [110]. A vaccine designed by coupling β-HCG to a an antibody directed against dendritic cells led to the generation of tumor-specific human leukocyte antigen (HLA) class I and class II-restricted T-cell responses, including cytotoxic T cells capable of killing human cancer cells expressing β-HCG [111]. In two recent phase I studies, the vaccine CDX-1307 consisting of β-HCG fused to an antibody against mannose receptor was administered either locally (intradermally) or systemically (intravenously) in 87 patients with advanced epithelial malignancies, including 27 breast cancer patients. An initial dose escalation of single-agent CDX-1307 was followed by additional cohorts of CDX-1307 combined with granulocyte-macrophage colony-stimulating factor (GM-CSF) and with Toll-like receptor (TLR) agonists to activate the antigen-presenting cells. CDX-1307 induced consistent humoral and T-cell responses to β-HCG when coadministered with TLR agonists. Greater immune responses and clinical benefit, including the longest duration of stable disease, were observed with immunization combined with local TLR agonists [112].

In other immunological approaches directed against ectopically expressed β-HCG, a monoclonal antibody against β-HCG was conjugated with cytotoxic drugs like curcumin or combined with Pseudomonas exotoxin in a recombinant immunotoxin. Both strategies in vitro succeeded in high killing rates of β-HCG -expressing cancer cells [47,113]. 

Thus, recent immunological strategies targeting cancer cell β-HCG either by vaccination or by targeted delivery of cytotoxic substances had promising results which clearly suggest that β-HCG might be a valuable target in cancer therapy.

## 3. Conclusions

Heterodimeric placental HCG and ectopically expressed β-HCG have opposing effects on breast cancer development. The reasons for these contrary effects are still controversial. One explanation could be the gylcosylation state of HCG, finding that hyperglycosylated β-subunits of HCG promote cancer cell invasion, growth and metastases. Another cause for the differing effect of HCG on breast cancer cells could be the fact that the *LHCGR* carries a large number of polymorphisms. As a consequence, sensitivity and plasma membrane expression can be changed, leading to different receptor activity. Moreover, *HCG* genes also carry many polymorphisms which leads to expression of different HCG types. All these points can cause opposing effects on breast cancer development and could explain controversial data at least partially. The protective effect of placental HCG on the mammary gland led to the hypothesis that mimicking pregnancy might be a strategy for breast cancer prevention. In contrast, the tumor-promoting β-HCG expressed in breast cancer cells seems to be a promising target for immunological approaches of breast cancer therapy. 

## Figures and Tables

**Table 1:** Pro- and antiapoptotic effects of human chorionic gonadotropin (HCG).

Proapoptotic Effects of HCG	Antiapoptotic Effects of HCG
**Downregulation of:**	**Downregulation of:**
• Estrogen receptor α ↓	• TGFβ ↓
• NF-κB ↓	• E-cadherin ↓
• AP-1 ↓	**Upregulation of:**
• PCNA ↓	• ERK1/2 ↑
• Ki67 ↓	• MMP-2 ↑
**Upregulation of:**	
• Inhibin ↑	
• β-casein ↑	
• Cytokeratin-18 ↑	
• E-cadherin ↑	
• p53 ↑	
• Caspase-9, -3 ↑	
• *TRPM2* ↑	
• ICE ↑	
• TGFβ↑	
• *BAX* ↑	
• *P21WAF1* ↑	

NF-κB: nuclear factor kappa B; TGF: transforming growth factor; PCNA: proliferating cell nuclear antigen; ERK1/2: extracellular signal–regulated kinase 1/2; MMP-2: matrix metallopeptidase 2; *TRPM2*: transient receptor potential cation channel subfamily M member 2; *BAX*: BCL2 associated X, apoptosis regulator; *P21WAF1*: cyclin-dependent kinase inhibitor p21.
